# Current Pharmacological Treatment of Type 2 Diabetes Mellitus in Undocumented Migrants: Is It Appropriate for the Phenotype of the Disease?

**DOI:** 10.3390/ijerph17218169

**Published:** 2020-11-05

**Authors:** Gianfrancesco Fiorini, Ivan Cortinovis, Giovanni Corrao, Matteo Franchi, Angela Ida Pincelli, Mario Perotti, Antonello Emilio Rigamonti, Alessandro Sartorio, Silvano Gabriele Cella

**Affiliations:** 1 Department of Clinical Sciences and Community Health, University of Milan, 20136 Milan, Italy; gianfrancesco.fiorini@grupposandonato.it (G.F.); ivan.cortinovis@unimi.it (I.C.); antonello.rigamonti@unimi.it (A.E.R.); 2 National Centre for Healthcare Research and Pharmacoepidemiology, 20126 Milan, Italy; giovanni.corrao@unimib.it (G.C.); matteo.franchi@unimib.it (M.F.); 3 Laboratory of Healthcare Research and Pharmacoepidemiology, Department of Statistics and Quantitative Methods, University of Milano-Bicocca, 20126 Milan, Italy; 4 Endocrinology and Diabetology Unit, San Gerardo Hospital, 20900 Monza, Italy; m.perotti@asst-monza.it (A.I.P.); angelaida.pincelli@noesim.it (M.P.); 5 Istituto Auxologico Italiano, IRCCS, Experimental Laboratory for Auxo-Endocrinological Research, 28824 Verbania, Italy; sartorio@auxologico.it

**Keywords:** type 2 diabetes, pharmacological treatment, undocumented migrants, diabetes phenotypes, ethnicity, complications of diabetes

## Abstract

Type 2 diabetes is increasingly recognized as a spectrum of metabolic disorders sharing chronic hyperglycaemia. In Europe, the continually growing number of migrants from developing countries could affect diabetes phenotypes. We evaluated a population of 426 Italians and 412 undocumented migrants. Using 17 variables (with the exclusion of ethnic origin) we performed a multiple component analysis to detect potential clusters, independently from ethnicity. We also compared the two groups to evaluate potential ethnicity associated differences. We found five clusters of patients with different disease phenotypes. Comparing Italians with undocumented migrants, we noted that the first had more often cardiovascular risk factors and neurologic involvement, while the latter had a higher frequency of diabetic ulcers and renal involvement. Metformin was used in a comparable percentage of patients in all clusters, but other antidiabetic treatments showed some differences. Italians were more often on insulin, due to a larger use of long acting insulin, and received a larger number of oral antidiabetics in combination. Pharmacological treatment of comorbidities showed some differences too. We suggest that type 2 diabetes should be considered as a spectrum of diseases with different phenotypes also in heterogeneous populations, and that this is not due only to ethnic differences.

## 1. Introduction

According to the International Diabetes Federation (IDF) the global prevalence of Diabetes (type 1 and type 2, either diagnosed or undiagnosed) has risen from 4.6% (151 million people) in 2000 to 9.3% (463 million people) in 2019 [1]. This trend is not expected to change, and estimates predict a rise to 642 million by 2040 [2].

Type 1 diabetes (T1D) is a well-defined disease. Type 2 diabetes (T2D), on the contrary, appears to represent a wide spectrum of metabolic disorders sharing chronic hyperglycaemia as a common feature. It is now clear that a classification more respondent to precision medicine criteria is needed, and attempts have been made to group T2D patients into different homogenous clusters [3,4]. This need for a better understanding of T2D phenotypes has practical implications for the pharmacological treatment of this disease. Indeed, the choice of treatment should no longer be exclusively based on a “glucocentric” vision. It should be patient-centred and consider also comorbidities and even social needs [5], as recommended by many authorities, including the American Diabetes Association (ADA) [6]. We also have to remember that costs for diabetes are high; for example, in Italy and in the United Kingdom they account for 10 % of the National Health Service expenditure [5,7]. This makes correct decisions even more compulsory: the better we know the target of our health strategies the better our interventions will be, both in terms of effectiveness and cost-effectiveness. It is known that phenotypic differences of T2D can be generated by several causes, as urbanization [8,9,10,11] and obesity [12,13]. However, ethnic differences may also play an important role, and this aspect has to be considered, since in the last few decades the phenomenon of migration from low-income countries is gradually changing the composition of the populations in most Western countries. These patients can be different: for example, it has been noticed that among African migrants, lean subjects may also have a high prevalence of T2D [8]. This could be due to other genetic factors, such as insulin resistance, which has an important role in determining T2D prevalence [9]. More in general, the impact of non-communicable diseases (NCD) in these migrant populations is relevant, though many aspects are still unclear [7,10,11]; certainly, ethnic differences may play a role [12]. This is true also for T2D; its prevalence in migrants is high [13,14], but again ethnic differences are relevant [12], with this disease being more frequent in people of South Asian, African and African-Caribbean family origin [5,15]. T2D is more likely to be poorly controlled in migrants [16], and can be less intensely treated in migrants than in natives [17]. This picture is made even more complicated by the fact that the information on NCD and their treatment in migrants is mainly obtained in documented migrants, for whom electronic health records are available. Undocumented migrants tend to escape epidemiologic surveys, since they have only occasional contacts with National Health Services (NHS). In this case, useful information can be obtained from datasets of non-governmental organizations (NGO) providing medical and pharmaceutical assistance to undocumented migrants. In previous studies, analysing this type of electronic records, we demonstrated that undocumented migrants have an important burden of NCD, including T2D [18] and that their diabetes has often a clinical presentation different from that of Italians [19]. Now, we designed this study to test the hypothesis that different phenotypic clusters of T2D also exist in undocumented migrants from in western populations. It would therefore be interesting to map these differences to specific clusters of patients, since T2D can show different clinical features in migrants from those in western populations [4]. Therefore, we consider many factors that can shape different clusters. Even more interesting would be to know if these patients receive appropriate pharmacological treatment, since this is fundamental to preventing and/or delaying harmful and resource-consuming complications. The present study begins to address these issues, which have a great significance for public health in consideration of the growing presence of migrants in Europe.

## 2. Methods

### 2.1. Patients

We studied 838 patients with T2D (412 undocumented migrants and 426 Italians). The former consisted of all of the diabetic patients cared for by a major NGO in Milan (Opera San Francesco, OSF) in 2018. An equivalent number of Italian patients was randomly chosen from among diabetic patients attending an outpatient diabetes clinic at a general hospital in Lombardy (San Gerardo Hospital, SGH) in the same year. Sixteen patients were excluded from further analysis because they had more than four missing variables.

OSF is one of the biggest NGOs operating in Italy. It delivers medical assistance to people living in poverty and receiving no assistance from the Italian NHS. This is made possible through the voluntary work of doctors running outpatient clinics for almost all specialties (including diabetology); medicines are dispensed for free to patients according to prescriptions after each consultation. The shifts of doctors are determined on the basis of a rota, comparable to that used in hospitals. Therefore, only seldom will the same doctor see the same patient twice, and this prevents prescription biases. It has also to be noted that all classes of antidiabetic medications are available in OSF; this does not happen in other smaller NGOs treating the same type of patients.

Patients of SGH attended an outpatient diabetes clinic in which they were seen by different doctors almost every time. Medicines were prescribed (and not dispensed); all the diabetologists of the clinic had the privileges required to prescribe all of them.

On the basis of their country of birth, migrants were grouped into the following ethnic groups: East Europe, North Africa, Sub-Saharan Africa, Latin America, West Asia, South-East Asia, China. The last three ethnic groups were defined on the basis a statistical analysis with the aim of obtaining more homogenous groups. For all patients, we recorded the main demographic data: age and gender, country of birth, marital status and education level. Health status indicators were also recorded and included: body mass index (BMI), presence of unhealthy lifestyles (smoking, alcohol), cardiovascular risk factors (obesity, hypertension, family history of ischaemic heart disease), presence of cardiovascular diseases. For each patient we gathered information on the following T2D related items: family history of diabetes, age of onset of the disease, hospital admissions for diabetes, presence of autoantibodies against glutamate decarboxylase (GADA) [20], presence of glycosuria, presence of ketonuria, levels of glycated haemoglobin (HbA1c), presence of complications (nephropathy, neuropathy, retinopathy, diabetic ulcers), and Q score. The latter is a composite indicator of how satisfactory metabolic control is in treated diabetics [21]. Of all these, three (marital status, education level and presence of autoantibodies) were almost always missing data for the migrants. Therefore, we did not use them for further analysis. However, in lean patients on insulin GADA, anti-IA and ICA were always determined to distinguish T2D from Latent Autoimmune Diabetes in Adulthood (LADA). Finally, from each database, we extracted pharmacological treatments for both groups of patients, as explained below.

### 2.2. Pharmacological Treatment

All pharmacological treatments were recorded for each patient. For those followed at SGH, this information was obtained directly from the outpatient dataset. For those followed at OSF, it was retrieved from the electronic database of the pharmacy. This database is built as follows. When a patient is seen, the doctor can prescribe him medicines that are then dispensed for free by the pharmacy, located in the same building of the outpatient clinic. This dispensation is recorded electronically with a light-pen, reading the barcode on each drug box. To make this data easier to analyse, we coded all prescriptions and dispensations according to the anatomical therapeutic chemical (ATC) classification [22], as previously described [18]. For insulin therapy, only five digits of the ATC codes were used. In this way all the insulins with ATC code A10AB were grouped as fast-acting insulins, while long-acting insulins could receive either the ATC code A10AD or A10AE. We recorded each oral antidiabetic with its seven-digit ATC code, even though they were usually analysed together, unless otherwise specified. For all patients, we also recorded the number of different oral antidiabetics they were on. Cardiovascular drugs were recorded with their seven-digit code. We then grouped all the codes beginning with C02 (antihypertensives), C07 (beta blocking agents), C08C (calcium channels blockers with mainly vascular effects) and C09 (agents acting on the renin-angiotensin system) under the heading “anti-hypertensive drugs”. Codes beginning with C10 (lipid modifying agents) formed the group of lipid-lowering drugs. All other drugs (in addition to antidiabetics and cardiovascular medicines) were recorded with their seven-digit ATC code.

### 2.3. Statistical Analysis

Percentage was used to describe each variable; Chi square test was used to evaluate the association between variables. A threshold of 0.05 for statistical significance has been adopted. Seventeen variables were used to create and study the profile of patients. Multiple correspondences analysis (MCA) was applied to find a low-dimensional graphical representation of the relationship among all these 17 variables. In the hyperspace dimensioned in relation to the number of variables (structural variables), the values of variables are the coordinates of a point that represents a patient. The centre of gravity of patients that have the same value for a variable can be reported in the hyperspace of variables and represents the position of that variable [23]. The distance between points, defined according to a chi-square metric, expresses the dissimilarities between points. When the points are close, the two patients or the two centres of gravity are very highly associated, namely, the subjects provided the same answer to the variables considered (in other words, the categories of a variable cannot distinguish between patients). MCA provides orthogonal factorial axes that are a linear combination of structural variables to describe the cloud of points. The information (variability) explained by each factorial axis was measured by an index suggested by Benzecri [24]. The variability explained by the first factorial plane is the sum of variability of the two first axes. Each factorial axis can be interpreted on the basis of the association with the variables that best describe it (higher percentage of variability explained by those variables). This makes it possible to describe the information present in a dataset without using any a priori interpretation.

Ethnicity was considered as a passive variable (this means that this variable did not contribute to define the factorial axes); in this way, it was possible to assess association between ethnicity and profiles of patients. The factorial coordinates of each subject were analysed with cluster analysis Ward algorithm. With this method there are no longer active or passive variables, but only the subjects. To assess the new variable “cluster” was associated with the variables accounting for the features of the subjects (including ethnicity) we ran a Chi square test. Differences exist among subjects belonging to different clusters. The strength of these differences is given by the results of Chi square tests. Five clusters were obtained and the patient profile of each cluster was studied to identify a peculiar profile of health condition. All the calculations were performed using SAS package version 9.4 (SAS Institute. Inc. Cary, NC, USA 2014).

### 2.4. Ethics

The study protocol was submitted to the Ethics Committee of the University of Milano–Bicocca. It was approved in the session of September 16, 2020 (protocol *n*. 564). All data were completely and permanently anonymized. For this reason, patient consent was waived. All procedures were in accordance with the ethical standards of the institutional and national research committee and with the 1964 Helsinki declaration and its later amendments or comparable ethical standards.

## 3. Results

### 3.1. Patient Characteristics

Table 1 shows the 17 variables we analysed with MCA and the variable “ethnicity” that we used as passive variable in the same analysis. The contribution of each variable to explaining every factorial axis (F1–F3) is also shown, together with the abbreviation of the variables’ names used in figures. F1 alone explains more than 60% of the cumulative variability of the sample; it appears to be strongly associated with age, age at diagnosis, neuropathy, unhealthy lifestyles and cardiovascular risk factors. It could be defined as the axis of patients’ age and health conditions associated with this variable. F2 is rather associated with glycosuria, ketonuria and BMI. On this axis, missing data are opposite to available data, and patients are differentiated on the basis of their BMI. F3 is associated with the following: glycosuria, retinopathy, age at diagnosis, duration of disease, HbA1c and Q score. It distinguishes patients on the basis of the severity of their pathology. Together, the first three axes explain around 83% of the whole variability; age, neuropathy and unhealthy lifestyles are the most important in characterising patients’ phenotypes. The weighted contribution to the explanation of the three axes, as shown in Table 1, is dependent on how much each variable counts in the explanation of an axis and how much this axis counts in the overall explanation of variability.

### 3.2. Patient Clusters

**Figure 1:**
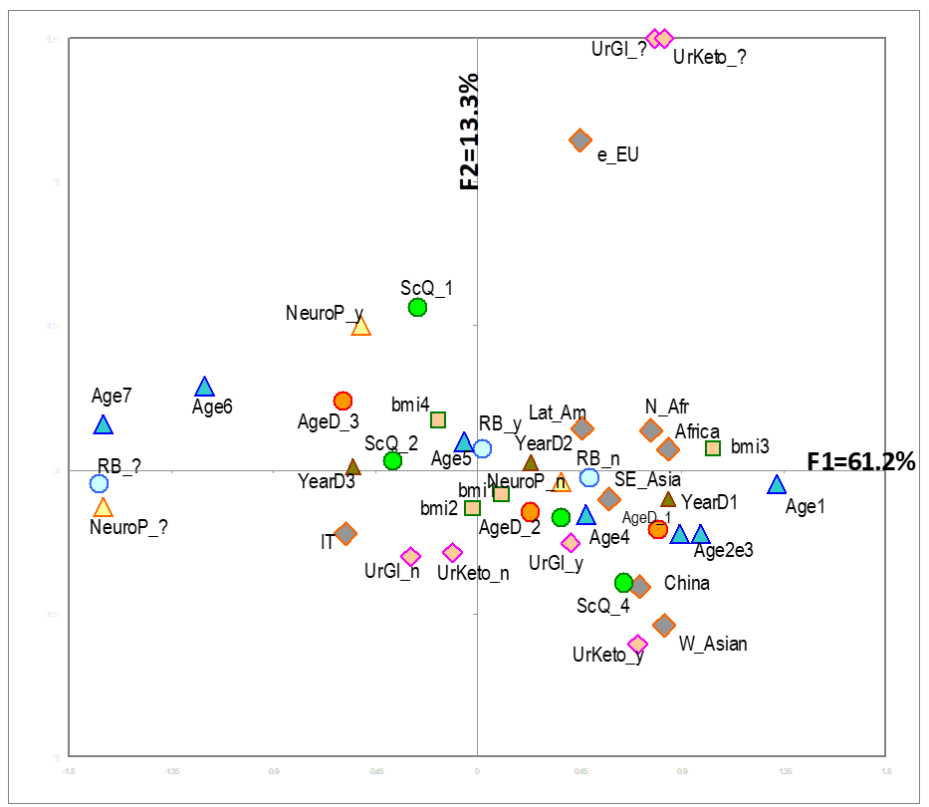
Graphic representation of the first factorial MCA plane. The variables with the greatest contribution to factorial axes are shown, together with the passive variable ethnicity. Abbreviations: Bmi: Body Mass Index; CVD: Cardiovascular Disease; FamD: Family history of Diabetes; NephP: Nephropathy; NeurP: Neuropathy; RB: Risk behaviors; RFCV: Risk factors for cardiovascular diseases; RetP: Retinopathy; ScQ: Score Q; UrGl: Glycosuria; UrKeto: Ketonuria.

**Figure 2:**
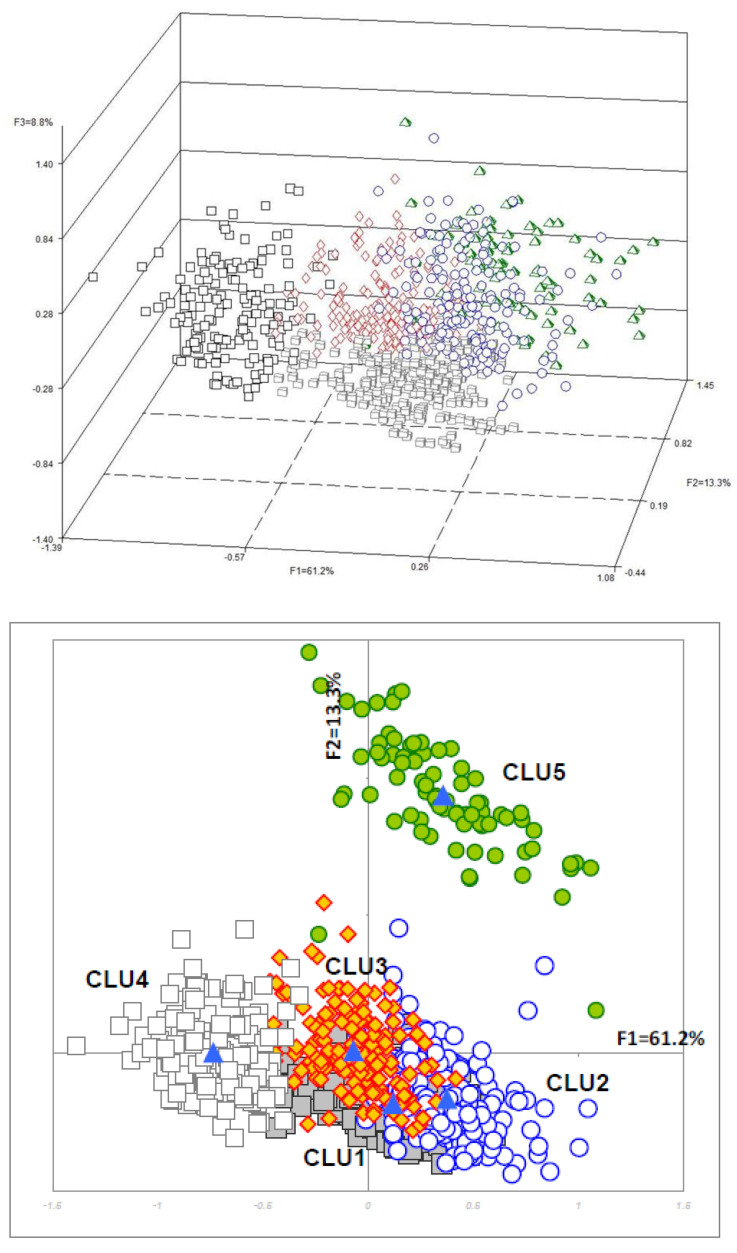
Graphic representation of patients in the five clusters. Above: on the first factorial plane. Below: in 3D. A blue triangle shows the centre of gravity of each cluster.

**Figure 3:**
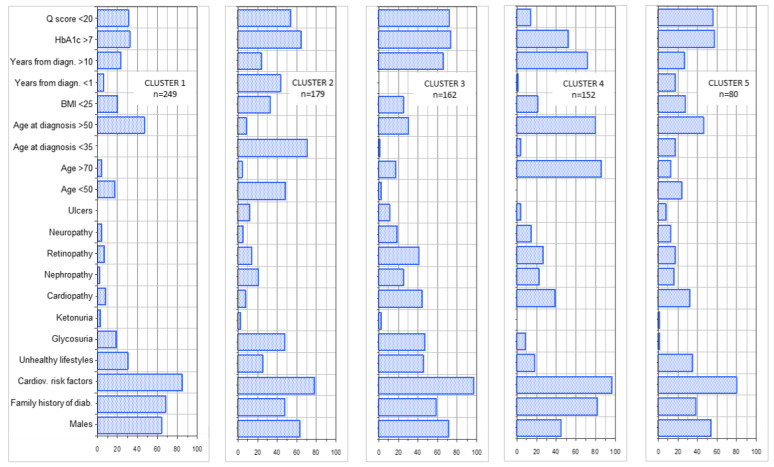
Composition of the five clusters (percentage of each of the 17 variables used in MCA).

**Figure 4:**
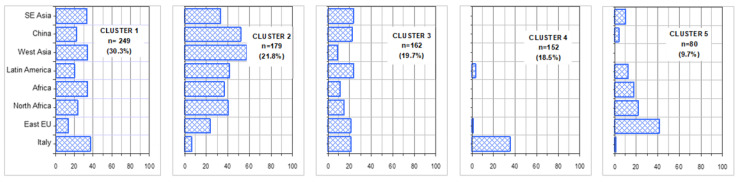
Distribution of the different ethnicities into the five clusters.

**Figure 5:**
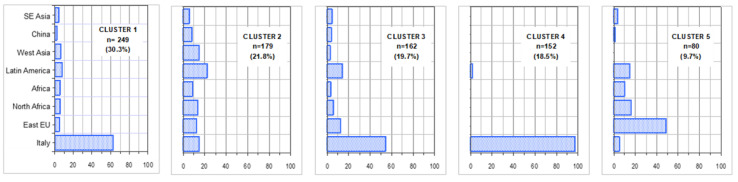
Ethnic composition of each cluster.

Figure 1 shows the variables more strongly associated with factorial axes on the first factorial plane, which explains almost 75% of total variability. The same figure shows also the variable ethnicity (passive variable in MCA). Italian patients appear to be more often associated with (i.e., closer to) older age and poorer health conditions (considering their position with respect to F1). Conversely (i.e., in the other hemi-space), we find patients of other geographical areas. For them, a distinction is evident between those with a higher proportion of missing data (Eastern Europe, North Africa, Latin America) and those with less missing data (Asia) and between those with different values of uric acid, ketonuria and younger age. Figure 2 shows the results of cluster analysis on coordinates of the first 4 factorial axes for each patient, i.e., almost 91% of overall variability of the sample. It also shows F1 with the projection of patients in different shape and colour according to the five clusters and a 3D representation of the same patients. The centre of gravity of each cluster is evidenced. Figure 3 gives a description of the patients of each cluster. The main features of the clusters were as follows. Cluster 1 (30.3% of patients)—It was mainly composed of Italians, though all other ethnic groups were present. The vast majority were aged between 50 and 70. More than 90% of the patients were free from nephropathy, cardiopathy, neuropathy and retinopathy. Around three quarters of these subjects had a Q score > 20. A total of 40% of patients with HbA1c <7% were in this cluster. Cluster 2 (21.8%)—It was the youngest cluster and that with the shorter duration of disease and the lowest age at diagnosis (<35 years in 71% of cases). Italians were a sharp minority. In 72% information on family history of diabetes was missing. More than 90% had no neurologic and cardiologic disease, while more than 20% had renal involvement. 42% of patients with diabetic ulcers were in this cluster. 64% had HbA1c levels greater than 7%. Cluster 3 (19.7%)—It was the cluster with the greatest percentage of males (70%). It also included the more compromised patients with over 70% of them having a Q score <20. 45% had unhealthy lifestyles. Diagnosis of diabetes had been made more frequently between 35 and 50 years of age and the majority of these patients had a disease history longer than 10 years. Retinopathy, cardiac disease and obesity were frequent (>40%). 65% had a history of disease longer than 10 years. HbA1c was >7% in 73% of patients. Cluster 4 (18.5%)—It was almost only made of Italians. Females were the majority. This was the cluster with the highest proportion of elderly patients (more than 70 years old). The diagnosis was made in advanced age and 71% had a disease history longer than 10 years. 79% had unhealthy lifestyles and 97% cardiovascular risk factors. In over 50% HbA1c was >7%. Cluster 5 (9.7%)—Nearly 50% were East-Europeans. This was the group with the highest percentages of missing data, especially for glycosuria (100%) and ketonuria (95%). More than 40% were obese. HbA1c was higher than 7% in more than a half of patients. Figure 4 shows the distribution of each ethnic group in the five clusters, while Figure 5 gives the composition, in terms of ethnic origin, of each cluster. Italians were the vast majority of cluster 4, represented about one half of cluster 1 and 3 and were very few in cluster 2 and almost absent in cluster 5.

### 3.3. Comparison between Italians and Undocumented Migrants

In general, comparing Italians with migrants as a whole, we noted that Italian patients tended to have more frequently factors of cardiovascular risk (401/421 vs. 329/409, *p* < 0.0001). The lowest percentage of patients with cardiovascular risk factor was in in the sub-Saharan group (66.7%). Neurologic involvement appeared to be more common in Italians (53/308 vs. 27/399, *p* < 0.0001), while migrants had more often diabetic ulcers (41/402 vs. 9/420, *p* < 0.0001) and renal involvement (80/394 vs. 51/410, *p* = 0.003). The latter appeared to be especially frequent in Latin Americans (26.7%) and East Europeans (19%). Though Italians were older than migrants (66 ± 10.3 years vs. 53.7 ± 11.6 years, *p* < 0.0001), they had a slightly shorter diabetes history (13.8 ± 10.3 years vs. 16 ± 18.8 years, *p* = 0.04).

### 3.4. Pharmacological Treatment

Pharmacological treatment of diabetes showed some differences between Italians and migrants. First, Italians were more often on insulin (211 vs. 152 *p* = 0.002). This difference was due to a greater number of patients on long acting insulin among Italians (205 vs. 139 *p* < 0.0001). On the contrary, the number of patients treated with fast acting insulin was the same in Italians and migrants (105 vs. 111 *p* = 0.5). For oral antidiabetic agents, too, the number of patients on treatment was greater among Italians (344 vs. 299 *p* = 0.003). Moreover, the mean number of different oral medications used by each patient was higher in Italians than migrants (1.3 ± 0.9 vs. 1.1 ± 0.8 *p* = 0.0001). Despite these differences, the number of patients receiving at least one pharmacologic treatment for diabetes was the same in both groups (Italians 406/425, migrants 394/413). Table 2 shows the percentages of patients on each pharmacological treatment in the five clusters. For diabetes, not unexpectedly metformin was the most used oral medication. Interestingly, however, a comparable percentage of patients in the different clusters was on metformin. On the contrary, use of insulin, both fast and long acting, was more frequent in clusters 3 and 4. Analysing the distribution of other pharmacological treatments, we found the same patterns for all of them, with a significant concentration in clusters 3 and 4. This was true for antihypertensives, lipid lowering drugs and anti-platelet agents. Information on other classes of medications was judged too incomplete; therefore, no further analysis was carried out. Finally, Table 3 shows the percentage of patients on each medication in the different ethnic groups.

## 4. Discussion

Before discussing our results, we have to underline a possible limitation of this study: our migrant patients were prevalent and not incident. Moreover, we cannot rule out the possibility that a longer duration of disease, previous therapy, or no therapy may have influenced the phenotype of their diabetes. All of this makes clustering less generalizable. However, our aim was to get a picture of the actual situation in our country and it suggests that T2D is a heterogeneous condition in all ethnic groups and therefore the choice of therapy should be based on the characteristics of individual patients. Moreover, it should be underlined that in our population, all migrants were undocumented. With these patients, controlled and randomised studies are virtually impossible to carry out.

After the above considerations, we think that some comments are worth to be made. First, our population of diabetic patients, made of Italians and undocumented migrants, appeared to be divided into five clusters on the basis of their clinical, anamnestic and demographic features, but not of ethnicity (passive variable). The fact that patients with T2D follow into different clusters is not surprising and has been demonstrated by other authors [4,25]. Complications of T2D follow a similar pattern and show differences among different clusters [26], and in turn the analysis of risk factors for T2D also generates different clusters with different prevalence and clinical features of T2D [27]. As specified above, our clusters were generated without considering ethnicity, which was then projected as a passive variable onto them; we also adopted the same procedure for pharmacological treatments.

The clusters were quite distinct. Patients in cluster 1 were adults with a satisfactory control of diabetes. Cluster 2 was mainly made of migrants and showed apparently contradictory features: young age, young age at diagnosis and short duration of disease, with a significant impact of diabetic ulcers and renal involvement. Renal involvement was reported to be evenly distributed in different clusters of diabetic patients by others [26], and indeed we found a comparable pattern in our study, with the exception of cluster 1, in which it was less frequent. However, it is strange that it affects young patients with a short duration of disease. The possibility that ethnic factors have some role cannot be ruled out. For example, it is known that chronic kidney disease is a significant problem in sub-Saharan Africans, both in their homeland and after migration [28]; however, a conclusion cannot be drawn, since too many factors are involved in the aetiopathogenesis of kidney disease, including even different psychosocial stressors [29]. Cluster 3 could be described as comprising patients with the worst metabolic control (low Q score and high HbA1c levels). Cluster 4 was characterised by the presence of older patients of female gender with unhealthy lifestyles and cardiovascular risk factors. It was made almost exclusively of Italian patients. Cluster 5 had the highest quantity of missing data, especially for glycosuria and ketonuria, and almost half was composed of East European patients. This is a somehow contradictory finding. The large amount of missing data could be due to poor adherence to treatment and follow-up; indeed, it is known that migrants have many difficulties in accessing health services [30,31], though attempts of implementing inclusive policies are made by many European countries [32]. However, this is not the case with our patients who, being undocumented migrants, receive medical assistance from an NGO, which is the same for all of them, including East Europeans. Moreover, we have previously reported that this ethnic group needs amounts of medicine for chronic diseases significantly higher than that needed by other ethnic groups [18]. An explanation for this contradictory observation could perhaps be found in the fact that OSF cannot provide all blood tests to all patients for free; therefore, this amount of missing data could be due to the fact that in this cluster we find the poorest patients, who cannot afford even small sums of money for their tests.

Comparing Italians with undocumented migrants as a whole, it appeared that Italians were older and had a slightly shorter duration of disease. Italians more often had cardiovascular risk factors and neurologic involvement. In migrants, on the contrary, diabetic ulcers and renal involvement were more frequent. These findings are in line with our previous observations [33] and raise some questions. It is known that different ethnic groups show different patterns of accumulation of chronic conditions [12], but other factors also play a role. One of these is gender, as we have demonstrated in a population of undocumented migrants [19]. Gender is also important when deciding how to deal with other chronic conditions such as, for example, arterial hypertension [34]. Poor socio-economic status could be even more important in determining the incidence of T2D and its phenotypic manifestations [35,36]. However, despite this, we have demonstrated that the pattern of chronic comorbidity still shows differences in different ethnic groups, after having taken into account socio-economic conditions [37].

The pharmacological treatment of diabetes showed some differences between Italians and migrants, too, though the number of patients on at least one medication was the same. When we projected the use of insulin and oral antidiabetics onto the five clusters, we found that it was more frequent in clusters 3 and 4. The composition of these two clusters in terms of ethnicity was different: about half of patients were Italian in cluster 3 (as in cluster 1) and the majority in cluster 4. Therefore, though we noticed some differences in insulin treatment between migrants and Italians, as also observed by others [22,38], it is unlikely that this is the most important factor. Indeed, clusters 3 and 4 were those with the longer duration of disease, cluster 3 was that with the worse glycaemic control, and cluster 4 was that with the oldest patients. In both clusters, obese patients were well represented, but this was true also for other clusters. Therefore, it is difficult to link the increased use of insulin in these two clusters to obesity related insulin resistance [39]. Other factors can also affect the different distribution of insulin treatment among clusters. For example, social or psychologic barriers to its use [40,41] could be important, and this in turn could be responsible for worse metabolic control [42]. Not unexpectedly, oral antidiabetic agents followed a comparable pattern of distribution, with the use of three or more medications being more frequent in cluster 3 and 4. Similar considerations to those made for insulin therapy could be made. On the contrary metformin was employed by an approximately comparable percentage of patients in clusters. This could be due both to ongoing recommendations [5,6] and to the acceptable safety of metformin, also in patients with renal failure [43]. Finally, lipid lowering drugs, anti-hypertensives and anti-platelet agents followed the same pattern: they were more frequently associated with cluster 3 and 4. A longer duration of disease and a higher frequency of cardiovascular diseases and of cardiovascular risk factors in these two clusters could be important for explaining this observation. Elderly age could also play a role in cluster 4. In cluster 3, two important factors could be the high percentages of males and the frequency of unhealthy lifestyles known to be important risk factors for cardiac diseases [44].

## 5. Conclusions

Our data are far from conclusive, but we can say that studying the features of T2D in a population made of western residents and undocumented migrants, we obtained distinct clusters of disease phenotypes. This is not different from what was observed by other researchers in more homogeneous populations [3,4]. This clustering appeared to be at least partially independent from racial/ethnical factors. Even more interesting is the fact that pharmacological treatment, with the exception of metformin, followed a distinct pattern of distribution among the clusters.

Our results can give further support to the opinion that a tailored, patient-centred approach to treatment should be used in all diabetic patients of all ethnic origins.

## Figures and Tables

**Table 1:** Composition of each variable in MCA. Percentages of explanation of the first three factorial axes and legends of abbreviations used in the figures are also given.

				Percentage of Contribution to the Explanation of the 3 First Factorial Axes (MCA)			Weighted Overall Contribution to the Explanation of the First 3 Axes
VARIABLE	Label	Abbreviation in Figures	% (*n* = 822)	F1	F2	F3	All Three Axes
TOTAL variability explained				61.2	13.3	8.8	83.3
Sex	female	female	39.4	0.9	0.7	0.0	0.6
	male	male	60.6				
Family history of diabetes	missing	FamD_?	6.6	3.6	3.7	4.2	3.1
	NO	FamD_*n*	32.0				
	YES	FamD_y	61.4				
Cardiovascular	missing	RFCV_?	0.4	5.5	0.1	1.0	3.5
risk factors	NO	RFCV_*n*	12.2				
	YES	RFCV_y	87.4				
Unhealthy lifestyles	missing	RB_?	16.4	17.7	0.1	0.0	10.9
	NO	RB_*n*	53.2				
	YES	RB_y	30.4				
Glycosuria	missing	UrGl_?	9.6	4.8	37.5	10.7	8.9
	NO	UrGl_*n*	63.4				
	YES	UrGl_y	27.0				
Ketonuria	missing	UrKeto_?	10.1	2.7	37.1	1.8	6.8
	NO	UrKeto_*n*	87.7				
	YES	UrKeto_y	2.2				
Cardiovascular	NO	CVD_*n*	76.8	2.7	5.1	2.1	2.5
Disease	YES	CVD_y	23.2				
Nephropathy	missing	NephP_?	2.2	1.0	2.5	9.1	1.7
	NO	NephP_*n*	81.9				
	YES	NephP_y	15.9				
Retinopathy	missing	RetP_?	5.0	1.8	1.5	10.0	2.2
	NO	RetP_*n*	75.2				
	YES	RetP_y	19.8				
Neuropathy	missing	NeurP_?	14.1	15.6	1.4	1.3	9.8
	NO	NeurP_*n*	75.9				
	YES	NeurP_y	10.0				
Ulcers	missing	Ulc_?	1.0	0.5	0.7	6.7	1.0
	NO	Ulc_*n*	92.9				
	YES	Ulc_y	6.1				
Age (years)	≤29	Age1	1.5	19.6	1.6	6.9	12.8
	30–39	Age2	3.6				
	40–49	Age3	13.6				
	50–59	Age4	29.3				
	60–69	Age5	29.3				
	70–79	Age6	16.1				
	≥80	Age7	6.6				
Age at Diagnosis (years)	≤35	AgeD1	18.2	8.6	1.9	14.9	6.8
	36–50	AgeD2	40.6				
	>50	AgeD3	41.2				
BMI	<18.5	bmi1	4.7	2.0	9.6	1.5	1.5
	18.5–24.9	bmi2	20.1				
	25–29.9	bmi3	37.0				
	≥30	bmi4	38.2				
Disease duration	<1	YearD1	13.4	7.3	0.1	9.5	5.3
(years from diagnosis)	2–9	YearD2	45.7				
	>10	YearD3	40.9				
Glycated	≤6.5	HbA_1	28.1	0.9	1.3	11.3	1.7
haemoglobin	6.6–7.0	HbA_2	18.2				
(%)	>7.0	HbA_3	53.7				
Q Score	≤10	ScQ_1	15.7	4.8	3.7	8.9	4.2
	11–20	ScQ_2	41.1				
	21–30	ScQ_3	31.4				
	31–40	ScQ_4	11.8				
Ethnicity	Italy	IT	51.3				
	East Europe	e_EU	11.6				
	North Africa	N_Afr	7.3				
	Sub-Saharan Africa	Africa	5.3				
	Latin America	Lat Am	11.8				
	West Asia	W Asian	5.7				
	China	China	3.3				
	South-East Asia	SE Asia	3.7				

Composition of each variable in MCA. Percentages of explanation of the first three factorial axes and legends of abbreviations used in the figures are also given. Abbreviations: Bmi: Body Mass Index; CVD: Cardiovascular Disease; FamD: Family history of Diabetes; NephP: Nephropathy; NeurP: Neuropathy; RB: Risk behaviors; RFCV: Risk factors for cardiovascular diseases; RetP: Retinopathy; ScQ: Score Q; UrGl: Glycosuria; UrKeto: Ketonuria.

**Table 2:** Percentage of subjects on each class of medication in the 5 clusters.

	Cluster 1 *n* 249	Cluster 2 *n* 179	Cluster 3 *n* 162	Cluster 4 *n* 152	Cluster 5 *n* 80
	%	%	%	%	%
Fast acting insulin	16.5	26.3	29.6	35.5	27.5
Long acting	30.1	36.9	53.7	52.6	37.5
Metformin	77.11	66.48	67.28	63.82	71.25
SGLPT 2 inhibitors	25.3	17.3	28.4	18.4	8.7
Other antidiabetic agents	24.1	25.7	24.7	28.9	30.0
Antihypertensives	55.8	40.9	67.9	82.2	62.5
Lipid lowering drugs	48.6	25.7	62.3	79.6	35.0
Anti-platelet agents	21.3	11.7	48.1	67.7	26.2

**Table 3:** Percentage of subjects on each class of medication in the different ethnic groups.

	Italy	East Europe	North Africa	Africa	Latin America	West Asia	China	South-East Asia
	%	%	%	%	%	%	%	%
Fast acting insulin	24.6	26.3	31.7	25.0	26.8	27.7	33.3	16.7
Long acting insulin	48.1	32.6	36.7	43.2	29.9	31.9	48.1	20.0
Metformin	75.6	69.5	51.7	59.1	69.1	70.2	29.6	80.0
SGLPT 2 inhibitors	29.6	8.4	5.0	13.6	17.5	21.3	7.4	13.3
Other antidiabetic agents	22.7	29.5	40.0	20.4	32.0	36.2	14.8	16.7
Antihypertensives	72.7	60.0	51.7	43.2	41.2	38.3	33.3	53.3
Lipid lowering drugs	76.1	36.8	15.0	25.0	24.7	14.9	18.5	16.7
Anti-platelet agents	49.3	35.8	11.7	11.4	9.3	6.4	25.9	10.0
